# ICTV Virus Taxonomy Profile: Kitaviridae 2026

**DOI:** 10.1099/jgv.0.002277

**Published:** 2026-08-05

**Authors:** Pedro L. Ramos-González, Juliana Freitas-Astúa, Jun-Min Li, Antonio Tiberini, Avijit Roy, Caixia Yang

**Affiliations:** 1 Applied Molecular Biology, Biological Institute, São Paulo, 04014-900, Brazil; 2 Embrapa Cassava and Fruits, Cruz das Almas-BA, 44380-000, Brazil; 3 Institute of Plant Virology, Ningbo University, Ningbo 315211, PR China; 4 Council for Agricultural Research and Economics, Research Centre for Plant Protection and Certification, Rome, 00156, Italy; 5 Molecular Plant Pathology Laboratory, Beltsville Agricultural Research Center, United States Department of Agriculture -Agricultural Research Service, Beltsville, MD 20705, USA; 6 Liaoning Key Lab. of Urban Integrated Pest Management and Ecological Security, Shenyang University, Liaoning 110044, PR China

**Keywords:** *Blunervirus*, *Cilevirus*, *Higrevirus*, ICTV Report, mite-borne viruses

## Abstract

The family *Kitaviridae* comprises plant-infecting viruses with segmented, positive-sense (+), ssRNA genomes, most of which are polyadenylated at the 3′-end. Members are classified into different genera based on their genome organization. Virions are short bacilliform, ellipsoidal or quasi-spherical and, in some species, enveloped. Transmission occurs via *Brevipalpus* mites (for members of the genera *Cilevirus* and *Higrevirus*) or eriophyid mites (for some members of the genus *Blunervirus*). Infections are typically localized due to the lack of or inefficient systemic viral movement. Kitavirids cause economically important plant diseases, including citrus leprosis. Phylogenetically, kitavirids are closely related to unclassified arthropod-infecting viruses. This is a summary of the International Committee on Taxonomy of Viruses (ICTV) Report on the family *Kitaviridae*, which is available at ictv.global/report/kitaviridae.

## Virion

**Fig. 1.**
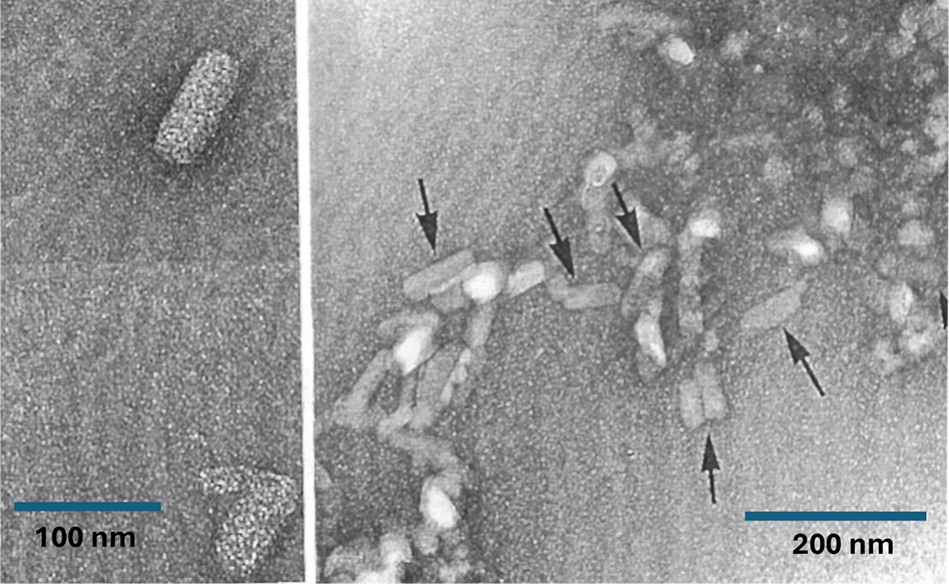
Electron micrograph of negatively-stained Citrus leprosis virus C (*Cilevirus leprosis*) showing bacilliiform particles (arrows) (Courtesy of Dr. Addolorata Colariccio, Instituto Biológico, SP, Brazil).

Kitavirid particles are bacilliform (Fig. 1), ellipsoidal or quasi-spherical, and, uniquely among plant-infecting alsuviricetes, virions of some species are lipid-enveloped (Table 1) [1, 2]. Kitavirids have segmented genomes, but whether particles contain more than one segment is unknown.

**Table 1.** Characteristics of members of the family *Kitaviridae*

Example	Citrus leprosis virus C (RNA1: DQ352194; RNA2: DQ352195), species *Cilevirus leprosis*
Virion	Short bacilliform particles, 25–70 nm in width and 100–120 nm in length, or ellipsoidal or quasi-spherical virions; particles of some species are lipid-enveloped
Genome	Two to five segments of polyadenylated positive-sense ssRNA, comprising 11–16 kb in total
Replication	Cytoplasmic, probably associated with the endoplasmic reticulum, involving the production of dsRNA intermediates
Translation	From genomic or subgenomic RNAs
Host range	Plants. Cileviruses and higreviruses are transmitted by *Brevipalpus* mites. Some blunerviruses are transmitted by eriophyid mites
Taxonomy	Realm *Riboviria*, kingdom *Orthornavirae*, phylum *Kitrinoviricota*, class *Alsuviricetes*, order *Martellivirales*; the family includes the genera *Cilevirus*, *Higrevirus* and *Blunervirus* and >25 species

## Genome

**Fig. 2.**
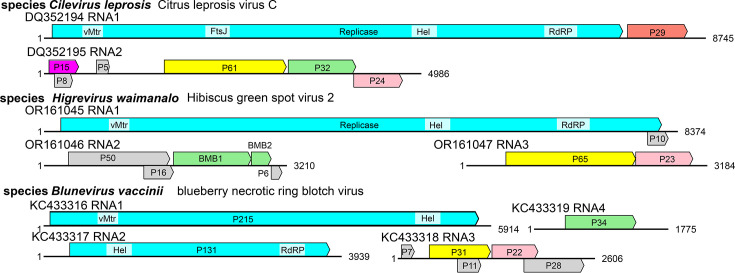
Genomic organization of representative kitavirids. ORF colours: replicase (cyan), structural glycoprotein (yellow), movement protein (green), structural protein (pink), coat protein (salmon), RNA silencing suppressor (magenta), and unknown function (grey). Domains within the replicase are methyltransferase (vMtr), FtsJ-like methyltransferase (FtsJ), helicase (Hel), and RNA-directed RNA polymerase (RdRP).

Kitavirid genomes comprise 11–16 kb of positive-sense ssRNA distributed across multiple segments, the number and organization of which vary by genus [1] (Fig. 2) . Kitavirid genomes encode a replicase containing the RNA-directed RNA polymerase (RdRP) domain (PF00978), and a transmembrane helix-containing protein (SP24), both of which are closely related to proteins encoded by unclassified arthropod negeviruses. Most kitavirid genomic RNAs have a poly-A tail at their 3′-end, preceded by non-coding regions that exhibit high sequence identity among isolates of the same species. A 5′-cap structure is presumed based on the presence of methyltransferase domains. Kitavirid genomes often contain numerous orphan ORFs and taxonomically-restricted ORFs of unknown function [1].

## Replication

Kitavirid replication occurs in the cytoplasm of infected cells, where dsRNA intermediates, presumed to be replication products, have been detected. Viral RNA synthesis is mediated by the replicase, which contains conserved methyltransferase, helicase and RdRP domains. Blunerviruses encode more than one helicase domain, and the RdRP and methyltransferase domains are encoded in separate polypeptides. For cileviruses, expression of downstream ORFs proceeds via a nested set of 3′-coterminal subgenomic RNAs. Viral replication and particle assembly occur in the cytoplasm, with virions accumulating within the lumen of the endoplasmic reticulum or in the perinuclear space. In cilevirus-infected cells, a budding-like process occurs at the endoplasmic reticulum adjacent to electron-dense viroplasms [1].

## Pathogenicity

Kitavirids induce localized, non-systemic infections. Disease symptoms typically manifest as chlorotic or necrotic lesions, often resulting in premature defoliation, fruit abscission and yield losses. The localized nature of kitavirid infections is generally associated with host defence responses, including a hypersensitive-like reaction that restricts viral replication. Viral factors, such as the cilevirus P61 protein, have been implicated in triggering this response and the associated cell death [3]. Cell-to-cell movement of most kitavirids is mediated either by a member of the 30K movement protein family or by a specialized mechanism known as the binary movement block [4, 5].

## Taxonomy

Current taxonomy: ictv.global/taxonomy. Genera within the family *Kitaviridae* are distinguished primarily by the genomic organization of their members, which form well-supported monophyletic clades in phylogenetic analyses based on the deduced amino acid sequences of the RdRP domains. Species demarcation within each genus is determined by the extent of serological relatedness, amino acid sequence identity across the viral proteome, natural host range, vector species and specific features of the transmission processcc

## Resources

Full ICTV Report on the family *Kitaviridae*: ictv.global/report/kitaviridae.

## Abbreviations

RdRP: RNA-directed RNA polymerase
